# Resveratrol Alleviated Intensive Exercise‐Induced Fatigue Involving in Inhibiting Gut Inflammation and Regulating Gut Microbiota

**DOI:** 10.1002/fsn3.70304

**Published:** 2025-05-23

**Authors:** Yuening Li, Qinsheng Li, Wenxiu Xu, Ruiqing Liu, Yanling Gong, Ming Li

**Affiliations:** ^1^ College of Competitive Sports Wuhan Sports University Wuhan China; ^2^ Physical Education Institute Taishan University Taian China; ^3^ College of Chemical Engineering Qingdao University of Science and Technology Qingdao China; ^4^ College of Physical Education and Health Linyi University Linyi China

**Keywords:** 16S rRNA, exercise‐induced fatigue, gut microbiota, intestinal mucosal barrier, resveratrol

## Abstract

Resveratrol (trans‐3,4′,5‐trihydroxystilbene, RES) is a stilbenoid naturally present in a variety of plants. Although there are several reports about its anti‐fatigue activity, its impact on intensive exercise‐induced fatigue and the underlying mechanisms are yet not well understood. In the present study, we established a swimming exercise protocol in mice that is similar to the fatigue condition induced by a long period of intensive exercise and explored the effect of RES on fatigue and the mechanisms from the perspective of intestinal injury and gut microbiota. The results revealed that RES significantly prolonged exhaustive swimming time in fatigued mice and improved the serum indexes associated with fatigue, including serum glucose, lactic acid (LA), urea nitrogen (BUN), lactate dehydrogenase (LDH), creatine kinase (CK), catalase (CAT), glutathione peroxidase (GSH‐Px), and glycogen storage in liver and muscle. Meanwhile, RES increased the expressions of ZO‐1, Occludin, and Claudin‐1, thereby enhancing intestinal barrier integrity and inhibiting mRNA expressions of tumor necrosis factor‐α (TNF‐α), interleukin‐6 (IL‐6), and interleukin‐1β (IL‐1β) in the colon, thereby improving the pathological injury in the colon. Importantly, RES modified gut microbiota dysbiosis by increasing the diversity of gut microbiota, regulating microbiota associated with inflammation and fatty acid metabolism at the phylum (*Bacteroidetes* and *Firmicutes*), family (*Erysipelotrichaceae*, *Enterobacteriaceae,* and *Prevotellaceae*), and genus ( *Brevundimonas diminuta* , *Coprobacillus*, *Megasphaera,* and *Lactobacillus*) levels, respectively. The results supplemented the anti‐fatigue mechanism for RES from the perspective of intestinal injury and gut microbiota. The detailed mechanisms and associated metabonomics analysis remain for further study.

## Graphical abstract

Resveratrol alleviated intensive exercise‐induced fatigue by inhibiting gut inflammation and enhanced the intestinal barrier integrity. Furthermore, Resveratrol modified gut microbiota dysbiosis by increasing the diversity of gut microbiota, regulating microbiota associated with inflammation and fatty acid metabolism at the phylum (Bacteroidetes and Firmicutes), family (Erysipelotrichaceae, Enterobacteriaceae and Prevotellaceae), and genus ( *Brevundimonas diminuta* , Coprobacillus, Megasphaera and Lactobacillus) levels, respectively.

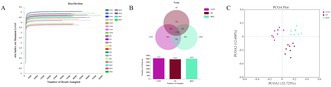

## 1 Introduction

Exercise‐induced fatigue (EF) is defined as a decrease of maximal voluntary muscle force induced by intense and prolonged exercise (Ament and Verkerke 2009). EF generally occurs in sports competition and physical training. The exhausting of energy sources and the accumulation of end products during EF result in the disturbance of the internal environment in the body (Tan et al. 2012). In addition, EF is also related to various physiological, pathological, and psychological factors, demonstrating a negative effect on other physiological functions including cardiac function and cognitive ability (Claessen and La Gerche 2016; Finsterer and Mahjoub 2014; Ma et al. 2018; Moore et al. 2012). It is reported that approximately half of the general population suffers from chronic fatigue sometime in their lifespans (Lei et al. 2016). Therefore, fatigue has become an important public health issue in urgent demand of novel supplemental food or therapeutic drugs. Of note, natural products show great advantages in the application of anti‐fatigue due to their safety and multiple activities (C. Luo et al. 2019; S. S. Zhou and Jiang 2019). It has promising potential to screen anti‐fatigue ingredients from natural products.

Resveratrol (trans‐3,4′,5‐trihydroxystilbene, RES) is a stilbenoid naturally present in a variety of plants including grapes, peanuts, soy, berries, and Itadori tea (Burns et al. 2002; Tian and Liu 2020). Due to its multiple pharmacological activities, RES has become one of the most studied polyphenols. After oral administration in a fasting state, RES is rapidly absorbed by the gastrointestinal tract and reaches peak plasma concentration (Cmax) within the first 30 min and 1.5‐2 h depending on doses (Huang, Lee et al. 2019). RES tends to be distributed in the brain, liver, intestine, and fat in animal bodies (Andres‐Lacueva et al. 2012). Due to its lipophilicity, RES exhibits a high volume of distribution (Vd) (Abd El‐Mohsen et al. 2006). The intestine and liver are recognized as the main metabolism sites for oral RES, demonstrating a concentration‐dependent biotransformation (X. T. Huang et al., 2019). Resveratrol and its metabolites are mainly eliminated through facial areas and urine (X. T. Huang et al., 2019). Studies have revealed that RES possesses antioxidative, anti‐inflammatory, antitumor, antiviral, anti‐obesity, anti‐aging, and antiapoptotic properties (Chen, Song et al. 2022; Cui et al. 2022; Molani‐Gol and Rafraf 2024; Rauf et al. 2018; D. D. Zhou et al. 2021). RES is also beneficial for hepatic, cardiac, brain, and nerve injuries, wound healing, glucose and lipid metabolism, etc. (Chupradit et al. 2022; Hecker et al. 2022; Rao et al. 2020; Q. Zhou et al. 2022; Zivarpour et al. 2022). Therefore, RES is widely applied in the food, cosmetic, and pharmaceutical industries (Giovinazzo et al. 2012; Ratz‐Łyko and Arct 2019; L. X. Zhang et al. 2021). Its potential application in food additives needs further study.

There are several reports concerning the anti‐fatigue effect of RES supplementation in recent years. Oral administration of RES to mice significantly prolonged the exhaustive swimming time and increased the grip strength, which might be associated with increasing energy utilization and decreasing serum lactate, ammonia, and creatine kinase (CK) (Wu et al. 2013). RES supplementation significantly increased aerobic capacity, tissue glycogen, and muscle hypertrophy in mice (Kan et al. 2018). Furthermore, RES supplementation during resistance exercise generated synergistic effects on the above performances (Kan et al. 2018). A clinical trial has revealed that supplementing RES in advance for young males significantly relieved muscle pain, increased exercise performance, and improved muscle damage induced by plyometric exercise‐induced muscle damage (C. C. Huang et al. 2021). All these studies suggested that RES possessed anti‐fatigue and performance improving effects. Otherwise, the detailed mechanisms for RES against fatigue are yet not well elucidated.

Therefore, we established an EF mice model and observed the anti‐fatigue effect of RES supplementation. Furthermore, the potential mechanisms of RES were explored from the perspective of intestinal injury and intestinal flora imbalance. The results are expected to supplement the possible mechanisms of RES against fatigue and provide a theoretical foundation for further development of RES.

## 2 Materials and Methods

### 2.1 Animals and Treatment

**FIGURE 1:**
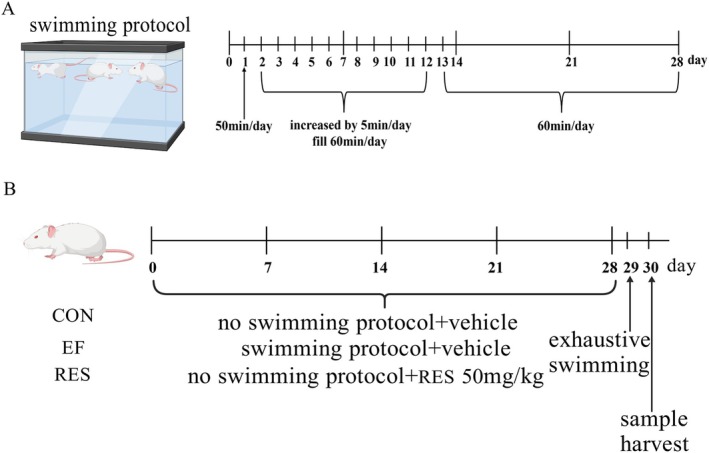
Swimming protocol (A) and mice grouping and treatment (B) in the present study.

Twenty‐four male ICR mice (8 weeks old, weighing 30–32 g), purchased from Qingdao Qinda Biotechnology Co. Ltd., were maintained at 24°C–26°C and 50%–60% humidity with a 12 h light/dark cycle. All mice were housed singly and fed a standard laboratory diet and distilled water ad libitum. After 1 week of acclimation, mice were randomly and equally assigned into 3 groups: control group (CON), exercise‐induced fatigue group (EF) and RES group (RES). The mice in RES group were given RES (50 mg/kg, Aladdin, Shanghai, China) by oral gavage once a daily for 28 days (Wu et al. 2013), while the mice in the CON and EF groups were given the same volume of solvent. The mice in the EF and RES groups were subjected to a swimming exercise protocol one hour after administration daily, which was designed to induce fatigue (Wang et al. 2023). Briefly, mice in the EF and RES groups were put into a water tank (50 cm × 60 cm × 60 cm) for swimming training with a water depth of 45 cm and a water temperature of 25°C. The swimming duration on the first day was 5 min and then increased by 5 min/day. On 12th day, the mice maintained swimming for 60 min. From then on, the mice conducted 60 min swimming duration in the following 16 days. The mice in the CON group were handled daily to mimic the disturbance caused by the experiment without swimming training. On the 29th day, all mice were put into a water tank for swimming, and the exhaustive swimming time was recorded when the mice sank into the water and failed to return to the surface within 10 s (Y. Chen, J. Wang, et al. 2022). On the 30th day, the samples were harvested after fasting overnight. The swimming protocol and mice grouping were demonstrated in Figure 1.

All animal experiments were approved by the Institutional Animal Care and Use Committee of Linyi University (No: LYU20240301) and conducted in accordance with the Guide for the Care and Use of Laboratory Animals.

### 2.2 Sample Collection

On the 30th day, the mice were anesthetized by intraperitoneal injection of 2% pentobarbital sodium (40 mg/kg) and the blood was collected from eyeballs to separate serum by centrifugation at 4°C, 3000 rpm for 10 min. Then, the mice were sacrificed to collect feces in the intestinal tract, as well as the liver, muscle, and colon. The liver and the muscle were prepared into 10% tissue homogenate with cold saline. The proximal colon was fixed in 4% polyformaldehyde to prepare paraffin sections, while the distal colon was stored at −80°C.

### 2.3 Serum Indexes and Tissue Glycogen Determination

Serum glucose, lactic acid (LA), urea nitrogen (BUN), lactate dehydrogenase (LDH), creatine kinase (CK), catalase (CAT), glutathione peroxidase (GSH‐Px), and glycogen in liver and muscle homogenates were measured using kits (Jiancheng bioengineering institute, Nanjing, China) and conducted following the instructions strictly.

### 2.4 Hematoxylin and Eosin (H&E) Staining

The colon tissue was embedded in paraffin after being fixed with polyformaldehyde, and then cut into slices with 2–4 μm. The slices were stained with hematoxylin and eosin (H&E, Beyotime, Shanghai, China), and then observed under light microscopy (Olympus, Tokyo, Japan) and photographed.

### 2.5 Immunohistochemistry

The paraffin slices were antigen repaired after dewaxing and hydration. Then the slices were sealed with sheep serum for 30 min. Subsequently, ZO‐1, Occludin, and Claudin‐1 primary antibodies (1:1000, Bioss, Beijing, China) were added and incubated overnight at 4°C, followed by operating with Polink‐2 plus Polymer HRP detection system (Bioss, Beijing, China) and DAB (Solarbio, Beijing, China) staining. The stained slices were observed under light microscopy (Olympus, Tokyo, Japan) and photographed. The images were quantified using Image J software (version 1.51j8, National Institutes of Health, USA).

### 2.6 Quantitative Real Time‐Polymerase Chain Reaction (qRT‐PCR)

The mRNA expressions of tumor necrosis factor‐α (TNF‐α), interleukin‐6 (IL‐6), and interleukin‐1β (IL‐1β) in the colon were determined by qRT‐PCR. Total RNA was extracted from colon tissue using the AG RNAex Pro kit (Agbio, Hunan, China) according to the manufacturer's protocol. Subsequently, cDNA was synthesized by reverse transcription using the BeyoRTII First Strand cDNA Synthesis Kit (Beyotime, Shanghai, China). qRT‐PCR was performed in a Quant StudioTM1 Real‐Time PCR Instrument (Thermo Fisher Scientific, Waltham, MA) using the ServicebioTM 2 × Universal Blue SYBR Green qPCR Master Mix (Servicebio, Wuhan, China). The mRNA expression level of the target gene was normalized to that of glyceraldehyde‐3‐phosphate dehydrogenase (GAPDH) using 2‐ΔΔCq. The primers were presented in Table 1.

**TABLE 1:** Primers for qRT‐PCR.

Gene	Forward sequence	Reverse sequence
TNF‐α	5’‐ACCCTCACACTCACAAACCA‐3’	5’‐GAGGCAACCTGACCACTCTC‐3’
IL‐6	5’‐GCCTTCTTGGGACTGATGCT‐3’	5’‐TGTGACTCCAGCTTATCTCTTGG‐3’
IL‐1β	5’‐TGCCACCTTTTGACAGTGATG‐3’	5’‐TTCTTGTGACCCTGAGCGAC‐3’
GAPDH	5’‐GGGGTCCCAGCTTAGGTTCA‐3’	5’‐CCCAATACGGCCAAATCCGT‐3’

### 2.7 16S rRNA Gene Sequencing

Bacterial DNA in the fecal samples was extracted to perform 16S rRNA gene sequencing and analysis. All the procedures were conducted by Sangon Biotech (Shanghai, China). The ASV denoising analysis was performed using the DADA2 algorithm to construct amplicon sequence variants (ASVs), with critical parameters set as follows: raw sequence quality filtering involved truncating forward/reverse reads at 240/200 bp, maximum expected error rate (maxEE) set to 2.0, and chimera detection using the consensus method. Taxonomic annotation was conducted based on the SILVA database (v138) full‐length 16S rRNA reference sequences, employing the Naive Bayes classifier within the QIIME2 framework for seven‐level taxonomic classification (from phylum to species), with a confidence threshold of 0.7. For diversity analysis, alpha diversity was assessed by calculating the Shannon index (species diversity), Observed OTUs (species richness), Chao1 (community abundance estimation), and Faith's PD (phylogenetic diversity), while beta diversity was evaluated using Bray–Curtis dissimilarity (community composition differences) and weighted/unweighted UniFrac distances (phylogenetic divergence), visualized via principal coordinate analysis (PCoA). Statistical analysis of intergroup differences was performed using PERMANOVA (Adonis algorithm) with a significance threshold of *p* < 0.05, and all analyses were completed on the QIIME2 platform (v2020.6).

### 2.8 Statistical Analysis

One‐way analysis of variance (ANOVA) followed by multiple comparison post hoc test was performed using GraphPad Prism 8.0 software (GraphPad Software, CA, USA). Tukey was used for post hoc test when normality and homogeneity of variance assumptions were met, while Dunnett's T3 was applied when not. *p* < 0.05 was considered statistical significance.

## 3 Results

### 3.1 RES Prolonged the Exhaustive Swimming Time in EF Mice

**FIGURE 2:**
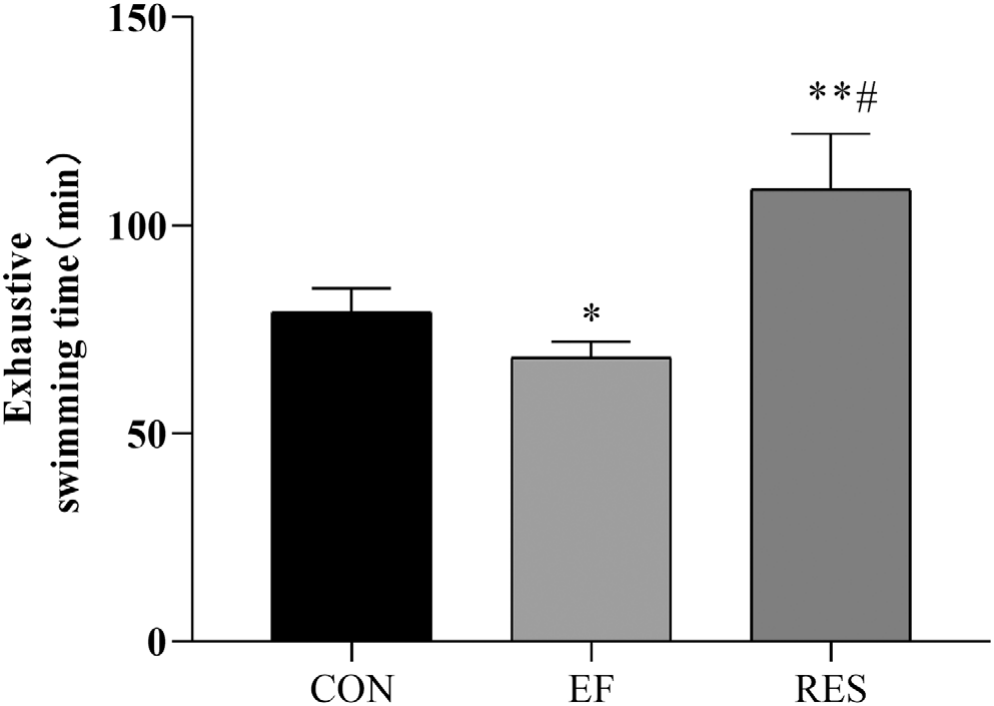
Effect of RES on exhaustive swimming time in EF mice. RES significantly prolonged exhaustive swimming time in EF mice, exhibiting an anti‐fatigue effect. Compared with CON group, *p < 0.05, **p < 0.01; Compared with EF group, #*p* < 0.01. *n* = 8.

As expected, the exhaustive swimming time decreased in EF mice when compared to CON mice (Figure 2, *p* < 0.05), indicating that a long period of intensive exercise reduced performance and resulted in fatigue. After oral administration of RES, the exhaustive swimming time was significantly prolonged when compared to EF mice and CON mice (Figure 2, *p* < 0.01), suggesting that RES exhibited an anti‐fatigue effect on EF mice and improved exercise performance.

### 3.2 RES Improved Serum Indexes in EF Mice

**FIGURE 3:**
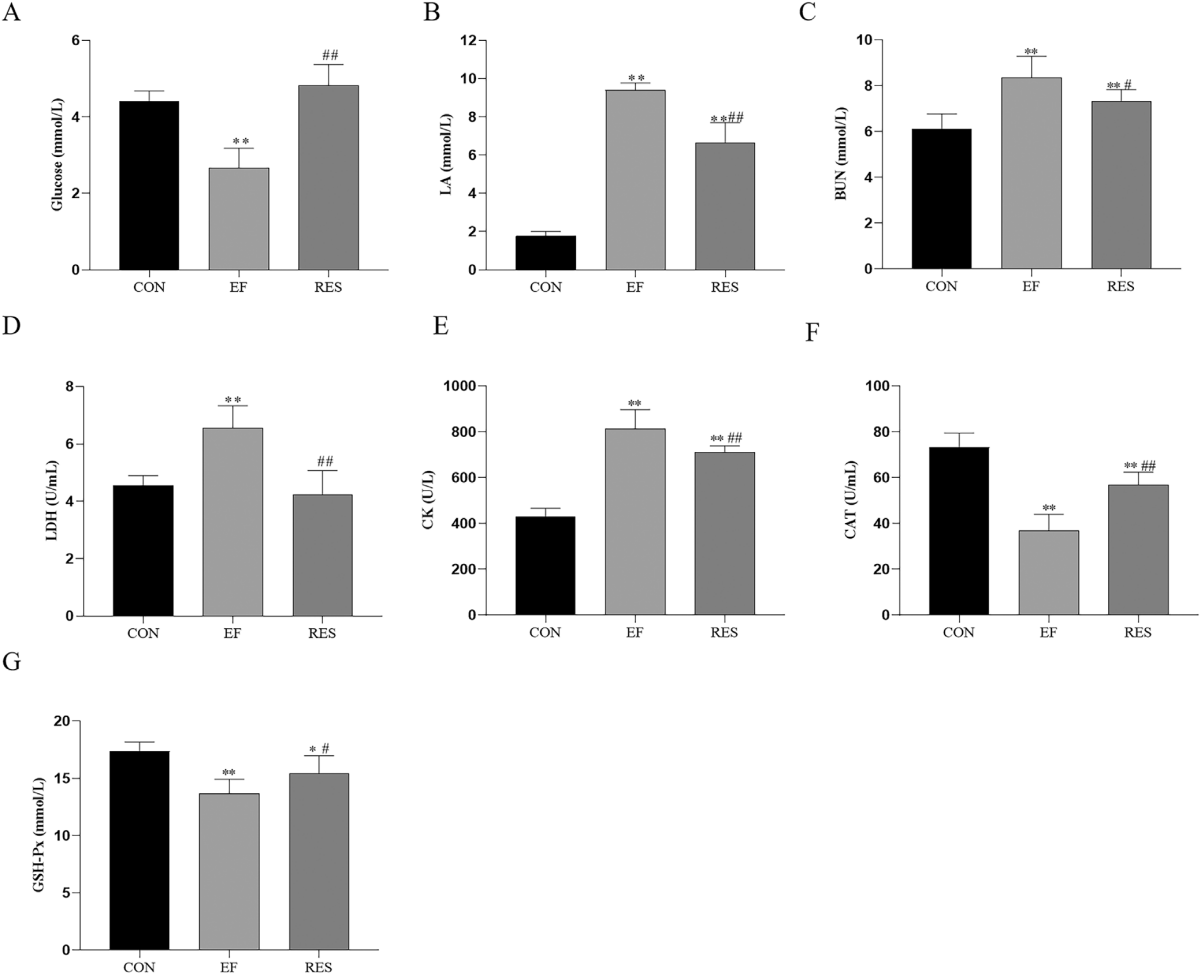
Effect of RES on serum indexes associated with fatigue in EF mice. RES significantly increased the levels of blood glucose, CAT, and GSH‐Px and decreased serum LA, BUN, LDH, and CK in EF mice, which was beneficial for the improvement of EF. Compared with CON group, *p < 0.05, **p < 0.01; Compared with EF group, #*p* < 0.05, ##*p* < 0.01. *n* = 8.

Several serum indices associated with EF were determined in the present study. As expected, the levels of blood glucose, CAT, and GSH‐Px significantly decreased while those of serum LA, BUN, LDH, and CK significantly increased in EF mice (Figure 3A–G, *p* < 0.05 or 0.01, comparing to CON group). After oral administration of RES, the levels of blood glucose, CAT, and GSH‐Px significantly increased while those of serum LA, BUN, LDH, and CK significantly decreased in EF mice (Figure 3A–G, *p* < 0.05 or 0.01, comparing to EF group). The results indicated that RES improved the above serum indices, which were beneficial for the improvement of EF.

### 3.3 RES Increased Liver Glycogen and Muscle Glycogen in EF Mice

**FIGURE 4:**
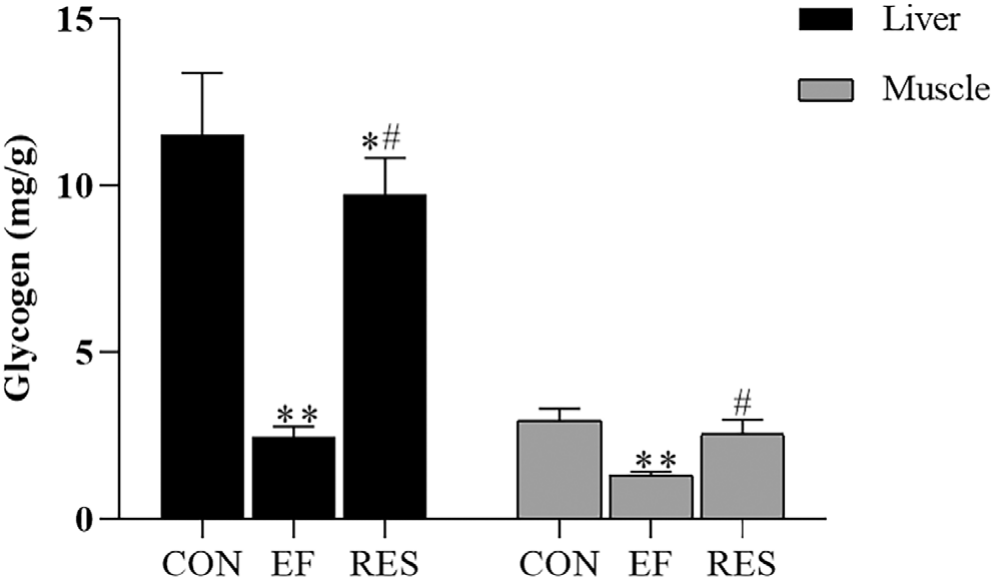
Effect of RES on liver glycogen and muscle glycogen in EF mice. RES significantly increased liver glycogen and muscle glycogen in EF mice, indicating that RES could increase glycogen storage to improve EF. Compared with CON group, *p < 0.05, **p < 0.01; Compared with EF group, #*p* < 0.01. *n* = 8.

As demonstrated in Figure 4, the glycogen levels in liver and muscle significantly decreased in EF mice (*p* < 0.01, comparing to CON mice), suggesting that exhaustive swimming resulted in glycogen consumption. After oral administration of RES, both liver glycogen and muscle glycogen significantly increased in EF mice (Figure 4, *p* < 0.01). The results revealed that RES could improve EF by increasing glycogen storage in liver and muscle.

### 3.4 RES Alleviated Intestinal Injury and Improved the Integrity of the Intestinal Mucosal Barrier in EF Mice

#### 3.4.1 RES Alleviated Intestinal Injury in EF Mice

**FIGURE 5:**
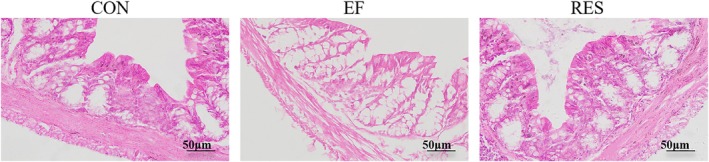
Effect of RES on pathological injury in colon of EF mice. RES significantly relieved colon injury induced by fatigue. Bars: 50 μm. *n* = 3.

Gastrointestinal injury induced by fatigue is an important concern that deserves more attention. Therefore, the pathological changes and the generation of inflammatory factors in the colon, as well as the integrity of the intestinal mucosal barrier, were explored in the present study. The H&E staining results revealed that the colon structure of CON mice exhibited no obvious abnormalities with the intact mucosal epithelium (Figure 5). However, there was observed an obvious pathological damage in the colon of EF mice (Figure 5). The intestinal mucosa was damaged, manifesting as bifurcation and atrophy of the intestinal recess. Ulceration, hyperplasia of connective tissue, reduction of goblet cells, and scattered inflammatory cell infiltration were observed in intestinal tissue (Figure 5). Of note, the above pathological damages of EF mice were significantly improved after oral administration of RES (Figure 5), suggesting that RES can relieve colon injury induced by fatigue.

#### 3.4.2 RES Inhibited Intestinal Inflammation in EF Mice

**FIGURE 6:**
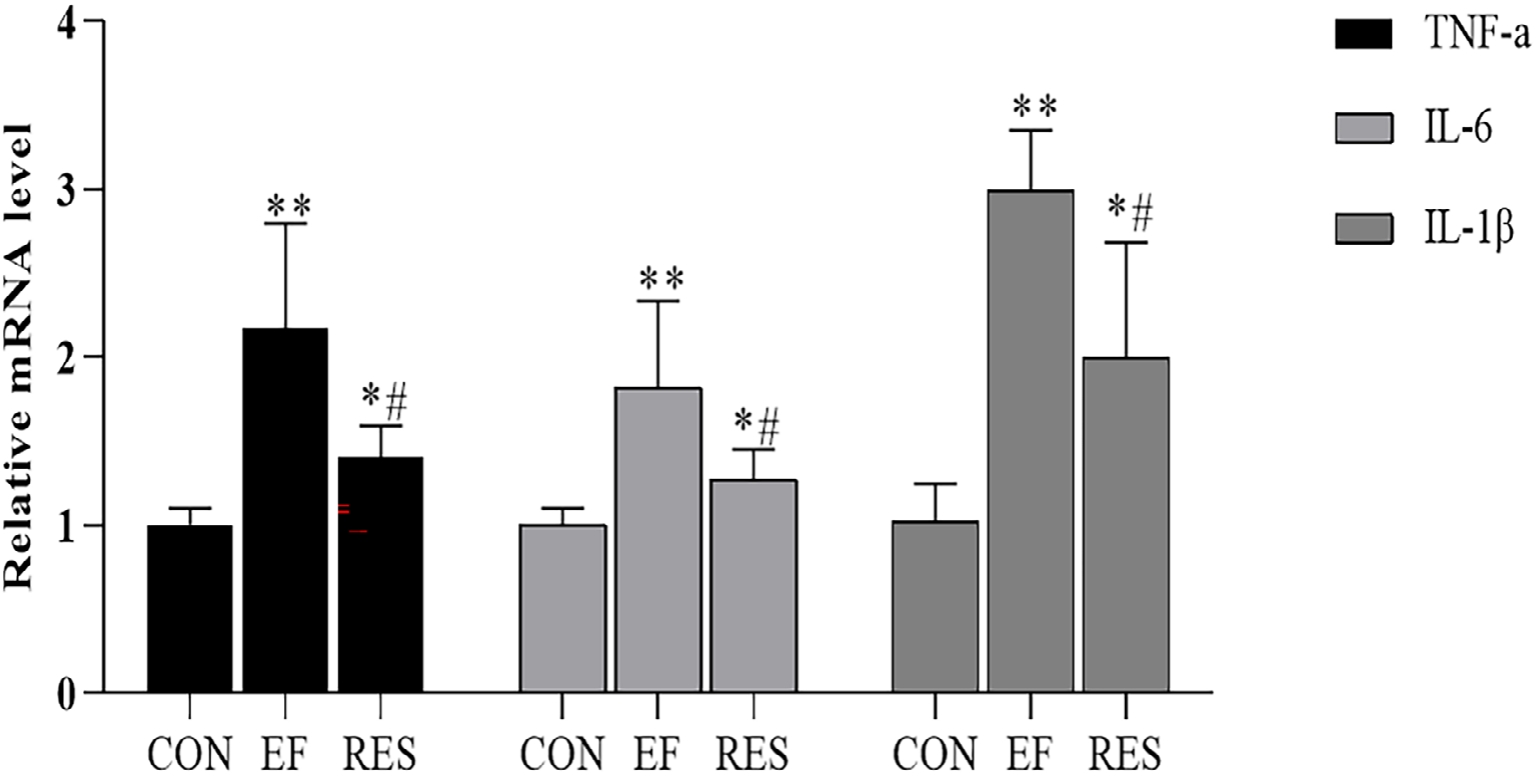
Effect of RES on mRNA expressions of inflammatory factors in colon of EF mice. RES significantly inhibited TNF‐α, IL‐6, and IL‐1β expressions in colon of EF mice, suggesting that RES inhibited the inflammatory reaction in the colon induced by fatigue. Compared with CON group, *p < 0.05, **p < 0.01; Compared with EF group, #*p* < 0.01. *n* = 3.

The qRT‐PCR results revealed that TNF‐α, IL‐6, and IL‐1β expressions significantly increased in EF mice when compared to CON mice (Figure 6, *p* < 0.01), suggesting that EF induced an inflammatory reaction in the colon. After oral administration of RES, TNF‐α, IL‐6, and IL‐1β expressions in EF mice decreased significantly, indicating that RES inhibited the inflammatory reaction in the colon induced by fatigue (Figure 6, *p* < 0.01, comparing to EF mice).

#### 3.4.3 RES Improved Intestinal Mucosal Barrier Integrity in EF Mice

**FIGURE 7:**
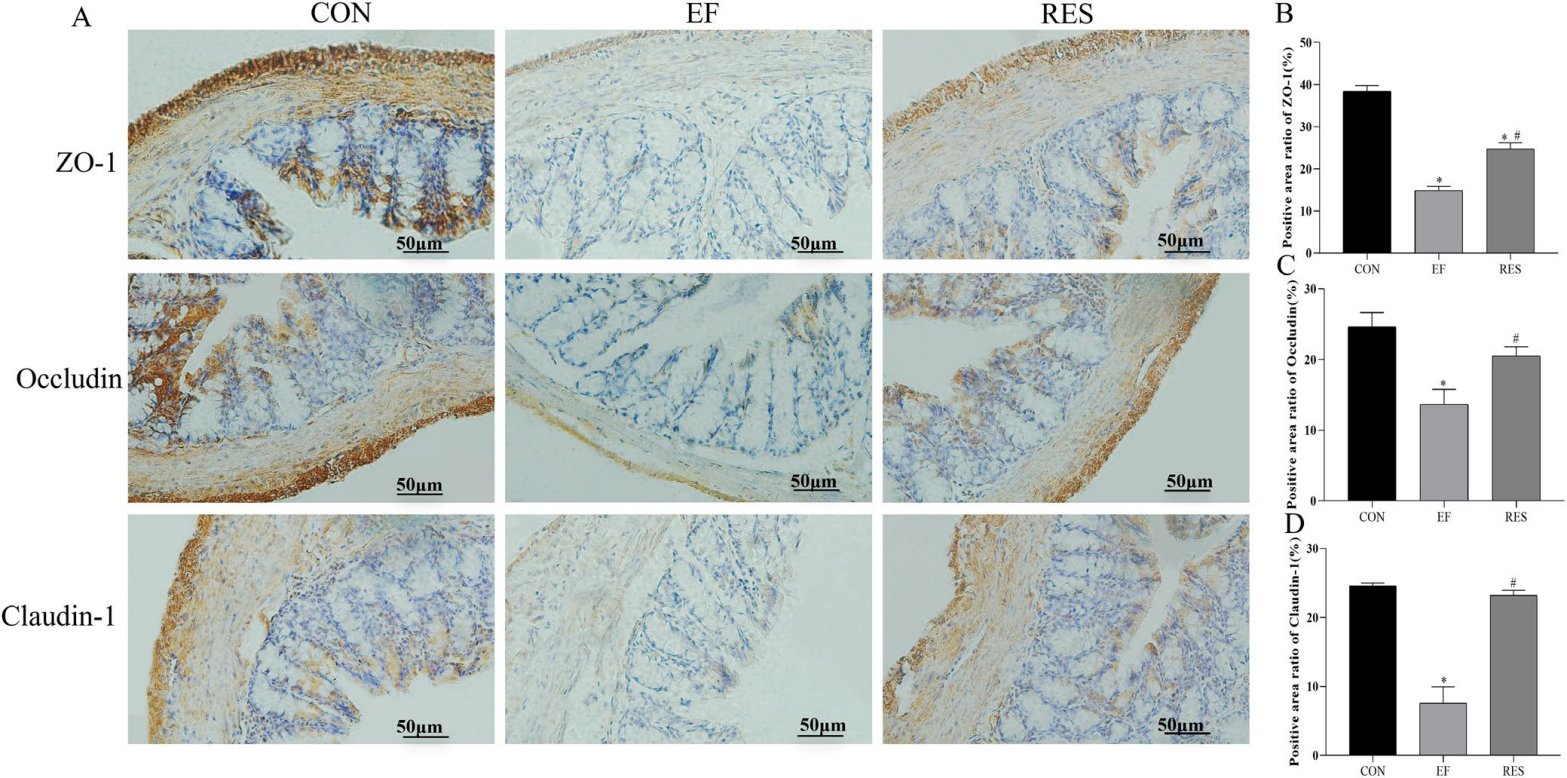
Effect of RES on expressions of ZO‐1, Occludin, and Claudin‐1 in colon mucosa of EF mice. RES significantly increased expressions of ZO‐1, Occludin, and Claudin‐1 (A for representative images, and B, C and D for quantitative analysis) in colon mucosa of EF mice, thereby maintaining the integrity of the intestinal mucosal barrier destroyed by fatigue. Bars: 50 μm. Compared with CON group, *p < 0.01; Compared with EF group, #*p* < 0.01. *n* = 3.

The pathological and inflammatory injury in the colon induced by fatigue might result in damage to the integrity of the intestinal mucosal barrier. Therefore, the expressions of tight junction proteins including ZO‐1, Occludin, and Claudin‐1 in the colon mucosa were determined by immunohistochemistry. As shown in Figure 7, there were abundant expressions of ZO‐1, Occludin, and Claudin‐1 that maintained the integrity of the intestinal mucosal barrier (Figure 7A). However, the integrity was damaged in EF mice, manifesting as the decreased expressions of ZO‐1, Occludin, and Claudin‐1 (Figure 7A–D, *p* < 0.01, comparing to CON group). Meanwhile, after oral administration of RES, expressions of ZO‐1, Occludin, and Claudin‐1 increased significantly (Figure 7‐D, *p* < 0.01, comparing to EF group), indicating an improvement in the integrity of the intestinal mucosal barrier destroyed by fatigue.

### 3.5 RES Modulated Gut Microbiota in EF Mice

#### 3.5.1 RES Increased the Diversity of Gut Microbiota in EF Mice

**FIGURE 8:**
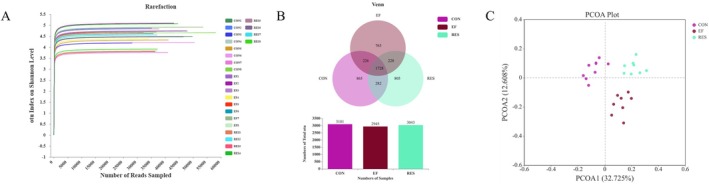
Effects of RES on diversity of gut microbiota in EF mice. The Shannon index (A), Venn analysis of the diversity differences (B), and PCoA score plot based on unweighted in the β‐diversity analysis (C) revealed that RES increased the diversity of gut microbiota in EF mice. *n* = 5.

The alpha diversity was demonstrated by the Shannon index curve, and each curve represents a sample (Figure 8A). The flattening of each sample's curve indicated that the sequencing data was sufficiently large to reflect the microbial diversity within the samples (Figure 8A). The Venn diagram showed that the gut microbiota diversity decreased in EF mice when compared to CON mice (Figure 8B), while it increased in RES mice when compared to EF mice (Figure 8B). According to the beta diversity analysis, the unweighted principal coordinate analysis (PCoA) exhibited significant differences in gut microbiota composition among the three groups (Figure 8C). The results revealed that RES could increase the diversity of gut microbiota in EF mice.

#### 3.5.2 RES Changed the Composition of Gut Microbiota in EF Mice

**FIGURE 9:**
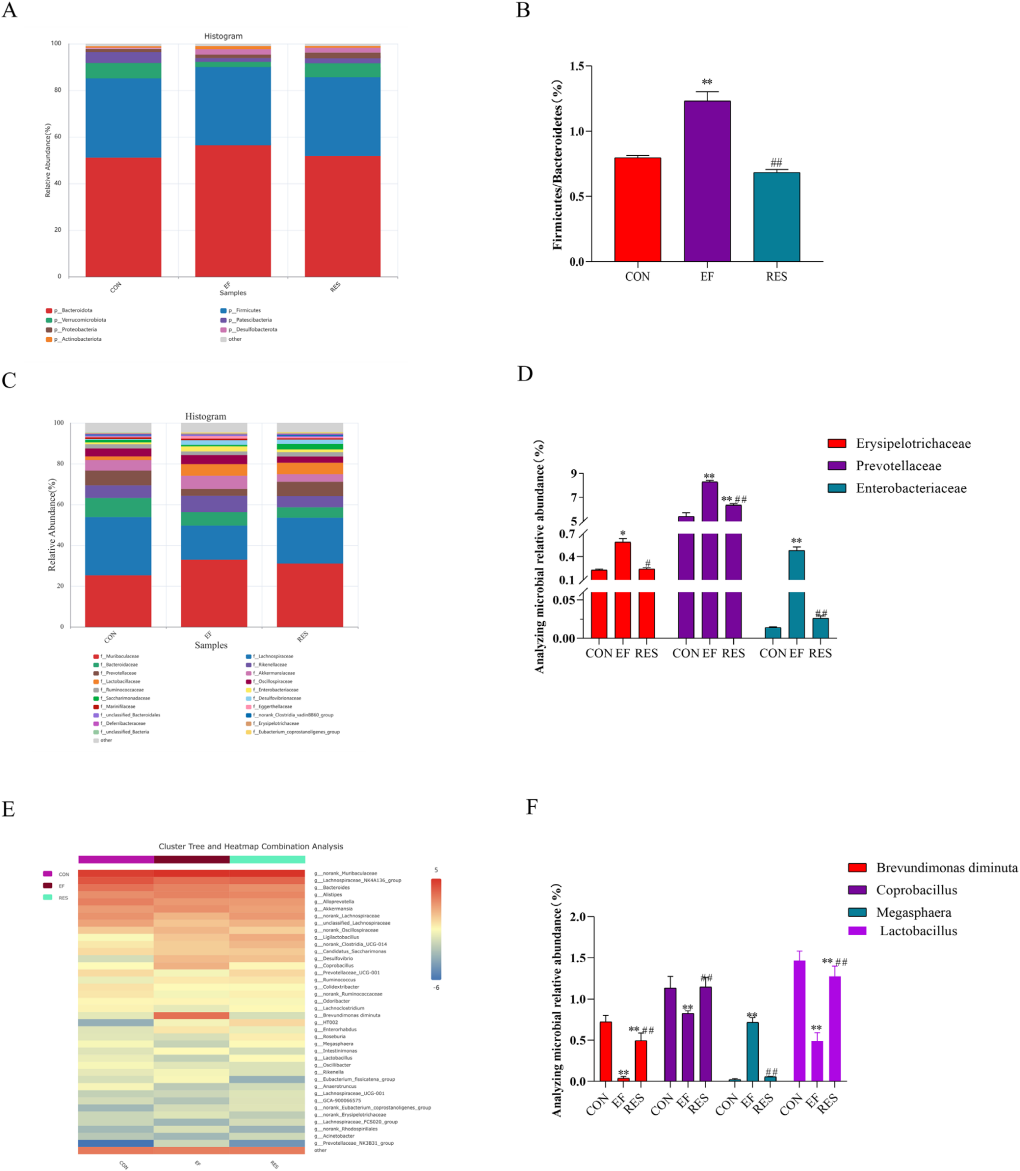
Effects of RES on enrichment of gut microbiota in EF mice at the phylum, family, and genus levels. RES decreased the Firmicutes to Bacteroidetes ratio at the phylum level (A for heatmap and B for bar graphs), decreased the enrichment of Erysipelotrichaceae and Enterobacteriaceae while increased that of Prevotellaceae at the family level (C for heatmap and D for bar graphs), and decreased the enrichment of *Brevundimonas diminuta* and Coprobacillus while increased that of Megasphaera and Lactobacillus at the genus level (E for heatmap and F for bar graphs). *n* = 5.

In the gut, the *Bacteroidetes* and *Firmicutes* are predominant at the phylum level. Therefore, our present study measured the relative composition of bacteria at the phylum level. The experimental results indicated that the *Firmicutes* to *Bacteroidetes* ratio significantly increased in EF mice when compared to CON mice (Figure 9A,D). Moreover, the *Firmicutes* to *Bacteroidetes* ratio tended to be similar in RES mice to that of CON mice (Figure 9A,D).

At the family level, it can be observed that the enrichment of *Erysipelotrichaceae* and *Enterobacteriaceae* increased sharply while *Prevotellaceae* decreased in EF mice. Otherwise, their changes were restored to normal levels after oral administration of RES (Figure 9B,E).

To further understand the changes in fecal microbial communities, a heatmap was used to display the key species with relatively high abundance (Figure 9C,F). Clearly, the gut microbiota of the three groups exhibited different levels at the genus level. The heatmap demonstrated that compared to CON mice, the enrichment of *Brevundimonas diminuta* and *Coprobacillus* in EF mice increased, while that of *Megasphaera* and *Lactobacillus* decreased. In RES mice, the enrichment of *Brevundimonas diminuta* , *Coprobacillus*, *Megasphaera*, and *Lactobacillus* tended to restore to the level of CON mice. In conclusion, RES restored the composition of gut microbiota induced by EF to the normal level.

## 4 Discussion

Although the anti‐fatigue effects of RES have been confirmed in the past, almost all literature evaluated the improvement of exercise performance and acute fatigue after exhaustive swimming. In our present study, we established a mice model of a long period of intensive exercise‐induced fatigue and observed the effect of simultaneous supplementation of RES on chronic fatigue. The results revealed that RES significantly prolonged exhaustive swimming time in mice with a long period of intensive exercise‐induced fatigue, showing anti‐fatigue activity. Meanwhile, the serum indexes associated with EF, including blood glucose, LA, BUN, LDH, CK, CAT, and GSH‐Px, as well as glycogen storage in the liver and muscle, were improved by RES. The above results demonstrated the anti‐fatigue activity of RES, in consistency with a previous report by Wu et al. (Wu et al. 2013). Unfortunately, there was no further exploration of the mechanism of RES in that report. In the next few studies, attempts were made to reveal the possible role of RES from an anti‐inflammatory and antioxidant perspective (Baltaci et al. 2016; Kan et al. 2018; Z. Xu et al. 2023). Of importance, it is the first time for our present study to explore the mechanisms of RES on EF from the perspective of intestinal injury and gut microbiota. Our present results revealed that RES significantly alleviated intestinal pathological injury, inhibited inflammatory reactions in the colon, and improved the integrity of the intestinal mucosal barrier induced by fatigue. Moreover, RES increased the diversity of gut microbiota and changed the composition of gut microbiota, which was disturbed by EF.

Swimming test is widely used as an ideal experimental animal model to evaluate the anti‐fatigue and activity for agents (C. J. Zhang et al. 2020). In most circumstances, weight‐loaded forced swimming test was employed to mimic acute fatigue conditions (Lee et al. 2022; X. Luo et al. 2023). In actual life, fatigue is generally caused by repetitive and sustained physical labor, resulting in internal environment disturbance (Chaudhuri and Behan 2004). In the present study, we established a swimming exercise protocol in mice that is similar to the fatigue condition induced by a long period of intensive exercise, with minor changes based on literature reports (Wang et al. 2023). In the swimming exercise protocol, mice were forced to swim daily for 28 d. The swimming duration on the first day was 5 min and increased by 5 min per day to 60 min on 12th day. From then on, the mice conducted a 60 min swimming duration in the following 16 d. As expected, fatigue developed after the long period of swimming exercise manifesting as changes in serum indexes and glycogen storage. Glucose is the main energy source for exercise and deficiency of glucose leads to the decrease of performance maintenance and results in fatigue (C. C. Huang et al. 2012). LA is a product of anaerobic glycolysis from carbohydrates in high intensive exercise, which results in the decrease of pH value both in blood and muscle tissue and leads to fatigue (Gibson and Edwards 1985; Hsiao et al. 2018). Therefore, serum LA level is a vital index to evaluate the exercise intensity and fatigue degree. Glycogen stored in the liver and muscle is another vital energy source that could supplement the consumption of glucose (Williams et al. 2013). Energy expenditure during intensive exercise results in the consumption of glycogen, which leads to fatigue (M. Xu et al. 2017). In consequence, increasing glycogen storage might improve exercise performance. BUN, a metabolic product of proteins, increases during high intensive exercise where protein metabolism is enhanced (X. Li et al. 2009). Therefore, BUN is used as a fatigue index, which is positively related to the degree of fatigue (W. C. Huang et al. 2015). Here we observed the decreased blood glucose, the increased LA and BUN, as well as the decreased glycogen in the liver and muscle in EF mice when compared with the CON mice. The results demonstrated that an EF model was successfully established by the swimming exercise protocol used in our present study. Meanwhile, RES significantly increased blood glucose, decreased LA and BUN, and increased liver glycogen and muscle glycogen, resulting in the alleviation of EF. As a consequence, the exhaustive swimming time was significantly prolonged in RES mice when compared with that in EF mice.

Several enzymes in the serum, such as LDH, CK, CAT, and GSH‐Px, are indexes to evaluate fatigue in clinical practice. LDH is an important glycolytic enzyme and is present in a variety of tissues, including muscle. The damaged muscle cells release LDH into the bloodstream, leading to an increase in serum LDH (Young et al. 2020). In addition to LDH, CK is another biomarker of muscle injury that is released by muscle cells into the bloodstream (Finsterer 2012). In addition, intensive exercise can induce oxidative stress damage by promoting the production of reactive oxygen species (Pingitore et al. 2015). The produced ROS could accelerate the oxidation of proteins, contributing to fatigue (Ruhee and Suzuki 2024). Therefore, scavenging ROS might be an efficient strategy to alleviate fatigue. CAT and GSH‐Px are key antioxidant enzymes that scavenge peroxides (X. Zhang et al. 2019). Here we observed the increased activities of serum LDH and CK as well as the decreased activities of serum CAT and GSH‐Px in EF mice, in line with the literature (Jeon et al. 2024; X. Zhang et al. 2019; Zhong et al. 2017). Moreover, oral administration of RES to EF mice significantly decreased the activities of serum LDH and CK and increased the activities of serum CAT and GSH‐Px, attributing to the alleviation of EF. There are several signaling pathways involved in the generation of antioxidants during EF alleviation, among which nuclear factor E2‐related factor 2 (Nrf2) is an important one (Zhao et al. 2023). During oxidative stress, Nrf2 translocates into the nucleus and then activates its downstream (antioxidant response element, ARE), which propels antioxidant enzymes (X. Zhang et al. 2019). Accumulating evidence has shown that RES enhances the Nrf2 signaling pathway to attenuate oxidative stress during various pathological processes (Chi et al. 2024; Farkhondeh et al. 2020; Shahcheraghi et al. 2023). Therefore, the anti‐fatigue effect of RES may be attributed to its activation of the Nrf2 signaling pathway, thereby upregulating the antioxidant enzymes such as CAT and GSH‐Px.

Gastrointestinal dysfunction is an important issue that needs attention during intensive exercise. In normal circumstances, an intestinal mucosal barrier prevents the diffusion of harmful substances including toxins, allergens, and pathogens from the intestinal lumen to the intestinal mucosa (Peterson and Artis 2014). In the intestinal mucosal barrier, tight junctions are important components that determine its physical barrier (Keita and Söderholm 2010). Tight junctions consist of a multiple transmembrane proteins including claudin, occludin, as well as intracellular plaque proteins, such as zonula occludens (ZO) and cingulin (Camilleri et al. 2012). Accumulating evidence has demonstrated that increased permeability induced by a destroyed intestinal mucosal barrier is associated with not only gastrointestinal diseases such as inflammatory bowel disease and irritable bowel syndrome, but also extragastrointestinal diseases including metabolic disease, cancer, anxiety, and depression (Cai et al. 2024; Chelakkot et al. 2018; Ciernikova et al. 2023; Crowley et al. 2024). Therefore, maintaining the integrity of the intestinal mucosal barrier might be effective for preventing or treating diseases. Stress causes the impairment of the intestinal mucosal barrier, as does the intensive exercise‐induced stress (Guo et al. 2022). As observed in our present study, the mRNA expression levels of TNF‐α, IL‐6, and IL‐1β in the colon significantly increased in EF mice, indicating that an inflammatory reaction occurred in the gut. Meanwhile, the expressions of tight junction proteins including ZO‐1, Occludin, and Claudin‐1 decreased significantly, resulting in the destruction of the integrity of the intestinal mucosal barrier. Of note, oral administration of RES to EF mice significantly increased the expressions of ZO‐1, Occludin, and Claudin‐1, thereby preventing the gut inflammatory reaction. The above results might be involved in the mechanisms of RES against EF. In addition, the inhibition of the inflammation‐signaling pathway might contribute to the alleviation of EF (Zhao et al. 2023). NF‐κB is an important indicator that mediates the inflammatory response. Upon inflammatory stimulus, NF‐κB translocates to the nucleus and results in the release of various inflammatory factors such as TNF‐α and IL‐1β (Mitchell and Carmody 2018). The NF‐κB signaling pathway is recognized as an essential molecular mechanism of RES to exert its anti‐inflammatory effects (Chen, Song et al. 2022). Therefore, in addition to modulating the intestinal barrier, the ability of RES to inhibit the NF‐κB pathway might also be associated with the suppression of gut inflammation, thereby alleviating fatigue.

Gut microbiota is another issue that has emerged in recent years. There are more than 100 trillion microorganisms dwelling in the human gastrointestinal tract, most of which are bacteria (J. Li et al. 2014). Approximately 50 bacterial phyla and about 100–1000 bacterial species comprise the gut microbiota (Adak and Khan 2019). The relatively stable gut microbiota is essential for maintaining the homeostasis of the host. Accumulating evidence has revealed that gut microbiota dysbiosis is involved in various diseases including metabolic disorders, cardiovascular diseases, neuropathy, psychological diseases, autoimmune diseases, tumors, etc. (Di Vincenzo et al. 2024). In recent years, the involvement of gut microbiota in EF has drawn intense attention. Moderate exercise is beneficial to health by modulating the gut microbiota. Otherwise, gut microbiota dysbiosis occurs under high intensive exercise, which in turn impacts the performance of the body and even results in disorders (Bermon et al. 2015). It suggests that gut microbiota might be a promising target to develop anti‐fatigue foods or drugs (Y. Li et al. 2023). Therefore, we detected the gut microbiota and aimed to explore the possible mechanisms of RES against EF. The results revealed that the diversity of gut microbiota decreased after EF while increased after simultaneous administration of RES. Changes in gut microbiota were also observed at the phylum, family, and genus levels, respectively. At the phylum level, the ratio of *Firmicutes* to *Bacteroidetes*, a marker of gut microbiota dysbiosis, increased in EF mice. The results confirmed gut microbiota dysbiosis induced by EF. At the family level, the enrichment of *Erysipelotrichaceae* and *Enterobacteriaceae* increased sharply while *Prevotellaceae* decreased in EF mice. *Erysipelotrichaceae* belongs to *Firmicutes*, which is generally related to inflammation‐related gastrointestinal diseases and metabolic disorders (Kaakoush 2015). The relative abundance of *Erysipelotrichaceae* is reported to be positively correlated to the TNF‐α level (Dinh et al. 2015). *Enterobacteriaceae*, a kind of proinflammatory bacterium, has been revealed to elevate in abundance in individuals with fatigue induced by cancer (Slack et al. 2024). Environmental and nutritional changes caused by inflammation in the gut lead to the increased abundance of Enterobacteriaceae (Garrett et al. 2010; Zeng et al. 2017). *Prevotellaceae* is a butyrate‐producing bacterium, and butyrate has anti‐inflammatory properties (Sitkin and Pokrotnieks 2019). The depletion of *Prevotellaceae* leads to intestinal barrier injury and gut microbiota dysbiosis (Y. Chen, Y. Liu, et al. 2022). As expected, RES decreased the enrichment of *Erysipelotrichaceae* and *Enterobacteriaceae* while increasing that of *Prevotellaceae*, which was related to the alleviation of EF. At the genus level, the enrichment of *Brevundimonas diminuta* and *Coprobacillus* in EF mice increased, while that of *Megasphaera* and *Lactobacillus* decreased. Similar changes in *Brevundimonas diminuta* , *Coprobacillus*, and *Megasphaera* were reported in mice with fatigue induced by intense exercise (N. Zhang et al. 2017). *Brevundimonas diminuta* is an emerging global opportunistic pathogen that has been reported in cases of bacteremia, pleuritis, keratitis, urinary tract infection, skin and soft tissue infection, empyema, and peritoneal dialysis‐associated peritonitis (Ryan and Pembroke 2018; Almuzara et al. 2012; Burch et al. 2021; Chandra et al. 2017). The abundance of *Coprobacillus* is reported to be correlated with the severity of IBS symptoms and fatigue (El‐Salhy 2023). Both *Megasphaera* and *Lactobacillus* can ferment LA, thereby accelerating the clearance of LA and improving EF (Cabral and Weimer 2024; Huang, Li et al. 2019). In EF mice treated with RES, the enrichment of *Brevundimonas diminuta* and *Coprobacillus* decreased and that of *Megasphaera* and *Lactobacillus* increased, resulting in the improvement of EF. In general, the changed gut microbiota in EF is almost involved in inflammation and fatty acid metabolism. Our results suggested that EF resulted in gut microbiota dysbiosis, attributing to oxidative stress, inflammation, and intestinal barrier dysfunction induced by EF (Y. Li et al. 2023). Surprisingly, RES restored gut microbiota dysbiosis, thereby alleviating EF. The harmful effects induced by EF, including inflammation, oxidative stress, and intestinal barrier damage, were closely associated with gut microbiota dysbiosis. On the other hand, gut microbiota dysbiosis, in turn, aggravates the above pathological damages (Y. Li et al. 2023; Y. Zhang et al. 2024). In consideration of the reciprocal causal relationship between gut microbiota dysbiosis and inflammation, it is difficult to address whether RES's effects on fatigue are directly mediated by gut microbiota or secondary to reduced inflammation. Both of them might contribute to the alleviation of EF by RES, and further study is in demand to reveal their detailed relationship. Due to the complexity of gut microbiota and its relationship with metabolites, metabonomics analysis is greatly needed to supplement the mechanisms of RES against EF. Of note, the fecal microbiota analysis does not fully represent mucosal or luminal communities. Therefore, multidisciplinary omics analysis, such as simultaneously conducting fecal microbiota analysis, intestinal mucosal microbiota analysis, host genome, and metabolome, might supply a more comprehensive understanding of the relationship between the gut microbiota and host health or disease. The dosage for mice in our present study was 50 mg/kg. According to the conversion factor of 9.1, the equivalent dosage for humans is 50/9.1 = 5.49 mg/kg. In a review concerning RES for the management of human health (Bo et al. 2016), the RES dosage used in multiple trials was 500 mg once or twice a day for more than 4 weeks. Therefore, the equivalent dosage of RES for humans in our present study is speculated to be safe and effective. Therefore, it is feasible for RES as a dietary supplement to alleviate EF. However, there is still a need to explore this in clinical trials.

## 5 Conclusion

The anti‐fatigue effect of RES on intensive exercise‐induced fatigue and its underlying mechanisms were explored in the present study. The results demonstrated that RES significantly prolonged exhaustive swimming time and improved the serum indexes associated with fatigue and glycogen storage. Meanwhile, RES increased the expressions of ZO‐1, Occludin, and Claudin‐1, thereby enhancing the intestinal barrier integrity and inhibiting the gut inflammatory reaction. Of importance, RES modified gut microbiota dysbiosis by increasing the diversity of gut microbiota, regulating microbiota associated with inflammation and fatty acid metabolism. It is the first time to reveal the anti‐fatigue mechanisms from the perspective of intestinal injury and gut microbiota. The detailed mechanisms and associated metabonomics analysis remain for further study.

## Author Contributions

Yuening Li: Methodology; Software; Data curation; Formal analysis; Investigation; Writing‐original draft; Writing‐review and editing; Conceptualization; Supervision; Qinsheng Li: Supervision; Data curation; Validation. Wenxiu Xu: Software; Data curation; Formal analysis. Ruiqing Liu: Supervision; Validation. Yanling Gong: Conceptualization; Methodology; Writing‐review and editing. Ming Li: Funding acquisition; Conceptualization; project administration; supervision; writing‐original draft; writing‐review and editing.

## Disclosure

Institutional Review Board Statement: All animal experiments were approved by the Institutional Animal Care and Use Committee of Linyi University and conducted in accordance with the Guide for the Care and Use of Laboratory Animals.

## Consent

The authors have nothing to report.

## Conflicts of Interest

The authors declare no conflicts of interest.

## Data Availability

The original contributions presented in the study are included in the article; further inquiries can be directed to the corresponding author.

## References

[fsn370304-bib-0001] Abd El‐Mohsen, M. , H. Bayele , G. Kuhnle , et al. 2006. “Distribution of [3H]Trans‐Resveratrol in Rat Tissues Following Oral Administration.” British Journal of Nutrition 96, no. 1: 62–70. 10.1079/bjn20061810. 16869992

[fsn370304-bib-0002] Adak, A. , and M. R. Khan . 2019. “An Insight Into Gut Microbiota and Its Functionalities.” Cellular and Molecular Life Sciences 76, no. 3: 473–493. 10.1007/s00018-018-2943-4. 30317530 PMC11105460

[fsn370304-bib-0003] Almuzara, M. N. , C. M. Barberis , C. H. Rodríguez , A. M. Famiglietti , M. S. Ramirez , and C. A. Vay . 2012. “First Report of an Extensively Drug‐Resistant VIM‐2 Metallo‐β‐Lactamase‐Producing *Brevundimonas Diminuta* Clinical Isolate.” Journal of Clinical Microbiology 50, no. 8: 2830–2832. 10.1128/jcm.00924-12. 22692741 PMC3421493

[fsn370304-bib-0004] Ament, W. , and G. J. Verkerke . 2009. “Exercise and Fatigue.” Sports Medicine 39, no. 5: 389–422. 10.2165/00007256-200939050-00005. 19402743

[fsn370304-bib-0005] Andres‐Lacueva, C. , M. T. Macarulla , M. Rotches‐Ribalta , et al. 2012. “Distribution of Resveratrol Metabolites in Liver, Adipose Tissue, and Skeletal Muscle in Rats Fed Different Doses of This Polyphenol.” Journal of Agricultural and Food Chemistry 60, no. 19: 4833–4840. 10.1021/jf3001108. 22533982

[fsn370304-bib-0006] Baltaci, S. B. , R. Mogulkoc , and A. K. Baltaci . 2016. “Resveratrol and Exercise.” Biomedical Reports 5, no. 5: 525–530. 10.3892/br.2016.777. 27882212 PMC5103661

[fsn370304-bib-0007] Bermon, S. , B. Petriz , A. Kajėnienė , J. Prestes , L. Castell , and O. L. Franco . 2015. “The Microbiota: An Exercise Immunology Perspective.” Exercise Immunology Review 21: 70–79. 25825908

[fsn370304-bib-0008] Bo, S. , V. Ponzo , G. Ciccone , et al. 2016. “Six Months of Resveratrol Supplementation has no Measurable Effect in Type 2 Diabetic Patients. A Randomized, Double Blind, Placebo‐Controlled Trial.” Pharmacological Research 111: 896–905. 10.1016/j.phrs.2016.08.010. 27520400

[fsn370304-bib-0009] Burch, J. , S. Tatineni , I. Enofe , and H. Laird‐Fick . 2021. “ *Brevundimonas diminuta* Coinfection as Source of Pyogenic Liver Abscess.” BML Case Reports 14, no. 5: e236235. 10.1136/bcr-2020-236235. PMC8117989 33975829

[fsn370304-bib-0010] Burns, J. , T. Yokota , H. Ashihara , M. E. Lean , and A. Crozier . 2002. “Plant Foods and Herbal Sources of Resveratrol.” Journal of Agricultural and Food Chemistry 50, no. 11: 3337–3340. 10.1021/jf0112973. 12010007

[fsn370304-bib-0011] Cabral, L. D. S. , and P. J. Weimer . 2024. “ *Megasphaera elsdenii* : Its Role in Ruminant Nutrition and Its Potential Industrial Application for Organic Acid Biosynthesis.” Microorganisms 12, no. 1: 219. 10.3390/microorganisms12010219. 38276203 PMC10819428

[fsn370304-bib-0012] Cai, Y. , W. Deng , Q. Yang , et al. 2024. “High‐Fat Diet‐Induced Obesity Causes Intestinal Th17/Treg Imbalance That Impairs the Intestinal Barrier and Aggravates Anxiety‐Like Behavior in Mice.” International Immunopharmacology 130: 111783. 10.1016/j.intimp.2024.111783. 38514921

[fsn370304-bib-0013] Camilleri, M. , K. Madsen , R. Spiller , B. Greenwood‐Van Meerveld , and G. N. Verne . 2012. “Intestinal Barrier Function in Health and Gastrointestinal Disease.” Neurogastroenterology and Motility 24, no. 6: 503–512. 10.1111/j.1365-2982.2012.01921.x. 22583600 PMC5595063

[fsn370304-bib-0014] Chandra, A. , A. Das , M. Sen , and M. Sharma . 2017. “ *Brevundimonas diminuta* Infection in a Case of Nephrotic Syndrome.” Indian Journal of Pathology & Microbiology 60, no. 2: 279–281. 10.4103/ijpm.Ijpm_679_15. 28631656

[fsn370304-bib-0015] Chaudhuri, A. , and P. O. Behan . 2004. “Fatigue in Neurological Disorders.” Lancet 363, no. 9413: 978–988. 10.1016/s0140-6736(04)15794-2. 15043967

[fsn370304-bib-0016] Chelakkot, C. , J. Ghim , and S. H. Ryu . 2018. “Mechanisms Regulating Intestinal Barrier Integrity and Its Pathological Implications.” Experimental & Molecular Medicine 50, no. 8: 1–9. 10.1038/s12276-018-0126-x. PMC6095905 30115904

[fsn370304-bib-0017] Chen, X. , X. Song , X. Zhao , et al. 2022. “Insights Into the Anti‐Inflammatory and Antiviral Mechanisms of Resveratrol.” Mediators of Inflammation 2022: 7138756. 10.1155/2022/7138756. 35990040 PMC9391165

[fsn370304-bib-0018] Chen, Y. , Y. Liu , Y. Wang , et al. 2022. “Prevotellaceae Produces Butyrate to Alleviate PD‐1/PD‐L1 Inhibitor‐Related Cardiotoxicity via PPARα‐CYP4X1 Axis in Colonic Macrophages.” Journal of Experimental & Clinical Cancer Research 41, no. 1: 1. 10.1186/s13046-021-02201-4. 34980222 PMC8722009

[fsn370304-bib-0019] Chen, Y. , J. Wang , Z. Jing , J. M. Ordovas , J. Wang , and L. Shen . 2022. “Anti‐Fatigue and Anti‐Oxidant Effects of Curcumin Supplementation in Exhaustive Swimming Mice via Nrf2/Keap1 Signal Pathway.” Current Research in Food Science 5: 1148–1157. 10.1016/j.crfs.2022.07.006. 35875345 PMC9304720

[fsn370304-bib-0020] Chi, F. , C. Cheng , M. Zhang , B. Su , Y. Hou , and G. Bai . 2024. “Resveratrol Targeting NRF2 Disrupts the Binding Between KEAP1 and NRF2‐DLG Motif to Ameliorate Oxidative Stress Damage in Mice Pulmonary Infection.” Journal of Ethnopharmacology 332: 118353. 10.1016/j.jep.2024.118353. 38762209

[fsn370304-bib-0021] Chupradit, S. , D. Bokov , M. Y. Zamanian , M. Heidari , and E. Hakimizadeh . 2022. “Hepatoprotective and Therapeutic Effects of Resveratrol: A Focus on Anti‐Inflammatory and Antioxidative Activities.” Fundamental & Clinical Pharmacology 36, no. 3: 468–485. 10.1111/fcp.12746. 34935193

[fsn370304-bib-0022] Ciernikova, S. , A. Sevcikova , B. Mladosievicova , and M. Mego . 2023. “Microbiome in Cancer Development and Treatment.” Microorganisms 12, no. 1: 1–28. 10.3390/microorganisms12010024. 38257851 PMC10819529

[fsn370304-bib-0023] Claessen, G. , and A. La Gerche . 2016. “Exercise‐Induced Cardiac Fatigue: The Need for Speed.” Journal of Physiology 594, no. 11: 2781–2782. 10.1113/jp272168. 27246544 PMC4887695

[fsn370304-bib-0024] Crowley, K. , Ł. Kiraga , E. Miszczuk , et al. 2024. “Effects of Cannabinoids on Intestinal Motility, Barrier Permeability, and Therapeutic Potential in Gastrointestinal Diseases.” International Journal of Molecular Sciences 25, no. 12: 1–33. 10.3390/ijms25126682. PMC11203611 38928387

[fsn370304-bib-0025] Cui, B. , Y. Wang , J. Jin , et al. 2022. “Resveratrol Treats UVB‐Induced Photoaging by Anti‐MMP Expression, Through Anti‐Inflammatory, Antioxidant, and Antiapoptotic Properties, and Treats Photoaging by Upregulating VEGF‐B Expression.” Oxidative Medicine and Cellular Longevity 2022: 6037303. 10.1155/2022/6037303. 35028009 PMC8752231

[fsn370304-bib-0026] Di Vincenzo, F. , A. Del Gaudio , V. Petito , L. R. Lopetuso , and F. Scaldaferri . 2024. “Gut Microbiota, Intestinal Permeability, and Systemic Inflammation: A Narrative Review.” Internal and Emergency Medicine 19, no. 2: 275–293. 10.1007/s11739-023-03374-w. 37505311 PMC10954893

[fsn370304-bib-0027] Dinh, D. M. , G. E. Volpe , C. Duffalo , et al. 2015. “Intestinal Microbiota, Microbial Translocation, and Systemic Inflammation in Chronic HIV Infection.” Journal of Infectious Diseases 211, no. 1: 19–27. 10.1093/infdis/jiu409. 25057045 PMC4326316

[fsn370304-bib-0028] El‐Salhy, M. 2023. “Intestinal Bacteria Associated With Irritable Bowel Syndrome and Chronic Fatigue.” Neurogastroenterology and Motility 35, no. 9: e14621. 10.1111/nmo.14621. 37246923

[fsn370304-bib-0029] Farkhondeh, T. , S. L. Folgado , A. M. Pourbagher‐Shahri , M. Ashrafizadeh , and S. Samarghandian . 2020. “The Therapeutic Effect of Resveratrol: Focusing on the Nrf2 Signaling Pathway.” Biomedicine & Pharmacotherapy 127: 110234. 10.1016/j.biopha.2020.110234. 32559855

[fsn370304-bib-0030] Finsterer, J. 2012. “Biomarkers of Peripheral Muscle Fatigue During Exercise.” BMC Musculoskeletal Disorders 13: 218. 10.1186/1471-2474-13-218. 23136874 PMC3534479

[fsn370304-bib-0031] Finsterer, J. , and S. Z. Mahjoub . 2014. “Fatigue in Healthy and Diseased Individuals.” American Journal of Hospice & Palliative Care 31, no. 5: 562–575. 10.1177/1049909113494748. 23892338

[fsn370304-bib-0032] Garrett, W. S. , C. A. Gallini , T. Yatsunenko , et al. 2010. “Enterobacteriaceae Act in Concert With the Gut Microbiota to Induce Spontaneous and Maternally Transmitted Colitis.” Cell Host & Microbe 8, no. 3: 292–300. 10.1016/j.chom.2010.08.004. 20833380 PMC2952357

[fsn370304-bib-0033] Gibson, H. , and R. H. Edwards . 1985. “Muscular Exercise and Fatigue.” Sports Medicine (Auckland, N.Z.) 2, no. 2: 120–132. 10.2165/00007256-198502020-00004. 3847097

[fsn370304-bib-0034] Giovinazzo, G. , I. Ingrosso , A. Paradiso , L. De Gara , and A. Santino . 2012. “Resveratrol Biosynthesis: Plant Metabolic Engineering for Nutritional Improvement of Food.” Plant Foods for Human Nutrition 67, no. 3: 191–199. 10.1007/s11130-012-0299-8. 22777386

[fsn370304-bib-0035] Guo, J. , X. Lou , W. Gong , et al. 2022. “The Effects of Different Stress on Intestinal Mucosal Barrier and Intestinal Microecology Were Discussed Based on Three Typical Animal Models.” Frontiers in Cellular and Infection Microbiology 12: 953474. 10.3389/fcimb.2022.953474. 36250050 PMC9557054

[fsn370304-bib-0036] Hecker, A. , M. Schellnegger , E. Hofmann , et al. 2022. “The Impact of Resveratrol on Skin Wound Healing, Scarring, and Aging.” International Wound Journal 19, no. 1: 9–28. 10.1111/iwj.13601. 33949795 PMC8684849

[fsn370304-bib-0037] Hsiao, C. Y. , Y. J. Hsu , Y. T. Tung , M. C. Lee , C. C. Huang , and C. C. Hsieh . 2018. “Effects of Antrodia Camphorata and *Panax ginseng* Supplementation on Anti‐Fatigue Properties in Mice.” Journal of Veterinary Medical Science 80, no. 2: 284–291. 10.1292/jvms.17-0572. 29276207 PMC5836765

[fsn370304-bib-0038] Huang, C. C. , M. C. Hsu , W. C. Huang , H. R. Yang , and C. C. Hou . 2012. “Triterpenoid‐Rich Extract From Antrodia Camphorata Improves Physical Fatigue and Exercise Performance in Mice.” Evidence‐Based Complementary and Alternative Medicine 2012: 364741. 10.1155/2012/364741. 22829854 PMC3398672

[fsn370304-bib-0039] Huang, C. C. , M. C. Lee , C. S. Ho , Y. J. Hsu , C. C. Ho , and N. W. Kan . 2021. “Protective and Recovery Effects of Resveratrol Supplementation on Exercise Performance and Muscle Damage Following Acute Plyometric Exercise.” Nutrients 13, no. 9: 3217. 10.3390/nu13093217. 34579095 PMC8469037

[fsn370304-bib-0040] Huang, W. C. , W. C. Chiu , H. L. Chuang , et al. 2015. “Effect of Curcumin Supplementation on Physiological Fatigue and Physical Performance in Mice.” Nutrients 7, no. 2: 905–921. 10.3390/nu7020905. 25647661 PMC4344567

[fsn370304-bib-0041] Huang, W. C. , M. C. Lee , C. C. Lee , et al. 2019. “Effect of *Lactobacillus Plantarum* TWK10 on Exercise Physiological Adaptation, Performance, and Body Composition in Healthy Humans.” Nutrients 11, no. 11: 1–15. 10.3390/nu11112836. PMC6893516 31752370

[fsn370304-bib-0042] Huang, X. T. , X. Li , M. L. Xie , et al. 2019. “Resveratrol: Review on Its Discovery, Anti‐Leukemia Effects and Pharmacokinetics.” Chemico‐Biological Interactions 306: 29–38. 10.1016/j.cbi.2019.04.001. 30954463

[fsn370304-bib-0043] Jeon, H. , K. Lee , Y. T. Kim , J. Y. Kim , J. J. Shim , and J. H. Lee . 2024. “Effect of HY7602 Fermented Deer Antler on Physical Fatigue and Antioxidant Activity in Mice.” International Journal of Molecular Sciences 25, no. 6: 3318. 10.3390/ijms25063318. 38542293 PMC10970475

[fsn370304-bib-0044] Kaakoush, N. O. 2015. “Insights Into the Role of Erysipelotrichaceae in the Human Host.” Frontiers in Cellular and Infection Microbiology 5: 84. 10.3389/fcimb.2015.00084. 26636046 PMC4653637

[fsn370304-bib-0045] Kan, N. W. , M. C. Lee , Y. T. Tung , C. C. Chiu , C. C. Huang , and W. C. Huang . 2018. “The Synergistic Effects of Resveratrol Combined With Resistant Training on Exercise Performance and Physiological Adaption.” Nutrients 10, no. 10: 1360. 10.3390/nu10101360. 30249003 PMC6212981

[fsn370304-bib-0046] Keita, A. V. , and J. D. Söderholm . 2010. “The Intestinal Barrier and Its Regulation by Neuroimmune Factors.” Neurogastroenterology and Motility 22, no. 7: 718–733. 10.1111/j.1365-2982.2010.01498.x. 20377785

[fsn370304-bib-0047] Lee, S. M. , Y. H. Kim , Y. R. Kim , et al. 2022. “Anti‐Fatigue Potential of *Pinus Koraiensis* Leaf Extract in an Acute Exercise‐Treated Mouse Model.” Biomedicine & Pharmacotherapy 153: 113501. 10.1016/j.biopha.2022.113501. 36076511

[fsn370304-bib-0048] Lei, H. , Y. Sun , Z. Luo , et al. 2016. “Fatigue‐Induced Orosomucoid 1 Acts on C‐C Chemokine Receptor Type 5 to Enhance Muscle Endurance.” Scientific Reports 6: 18839. 10.1038/srep18839. 26740279 PMC4703980

[fsn370304-bib-0049] Li, J. , H. Jia , X. Cai , et al. 2014. “An Integrated Catalog of Reference Genes in the Human Gut Microbiome.” Nature Biotechnology 32, no. 8: 834–841. 10.1038/nbt.2942. 24997786

[fsn370304-bib-0050] Li, X. , H. Zhang , and H. Xu . 2009. “Analysis of Chemical Components of Shiitake Polysaccharides and Its Anti‐Fatigue Effect Under Vibration.” International Journal of Biological Macromolecules 45, no. 4: 377–380. 10.1016/j.ijbiomac.2009.07.005. 19643126

[fsn370304-bib-0051] Li, Y. , J. Li , F. Xu , et al. 2023. “Gut Microbiota as a Potential Target for Developing Anti‐Fatigue Foods.” Critical Reviews in Food Science and Nutrition 63, no. 18: 3065–3080. 10.1080/10408398.2021.1983768. 34592876

[fsn370304-bib-0052] Luo, C. , X. Xu , X. Wei , et al. 2019. “Natural Medicines for the Treatment of Fatigue: Bioactive Components, Pharmacology, and Mechanisms.” Pharmacological Research 148: 104409. 10.1016/j.phrs.2019.104409. 31446039

[fsn370304-bib-0053] Luo, X. , W. Liu , B. Zheng , et al. 2023. “Sea Cucumber Peptides Positively Regulate Sexual Hormones in Male Mice With Acute Exhaustive Swimming: Possibly Through the ca(2+)/PKA Signaling Pathway.” Food & Function 14, no. 22: 10188–10203. 10.1039/d3fo03031h. 37909356

[fsn370304-bib-0054] Ma, J. , H. Chen , X. Liu , L. Zhang , and D. Qiao . 2018. “Exercise‐Induced Fatigue Impairs Bidirectional Corticostriatal Synaptic Plasticity.” Frontiers in Cellular Neuroscience 12: 14. 10.3389/fncel.2018.00014. 29422839 PMC5788965

[fsn370304-bib-0055] Mitchell, J. P. , and R. J. Carmody . 2018. “NF‐κB and the Transcriptional Control of Inflammation.” International Review of Cell and Molecular Biology 335: 41–84. 10.1016/bs.ircmb.2017.07.007. 29305014

[fsn370304-bib-0056] Molani‐Gol, R. , and M. Rafraf . 2024. “The Anti‐Obesity Effects of Resveratrol on the 3T3‐L1 Adipocytes.” International Journal for Vitamin and Nutrition Research 94, no. 3–4: 252–263. 10.1024/0300-9831/a000784. 37248954

[fsn370304-bib-0057] Moore, R. D. , M. W. Romine , P. J. O'Connor , and P. D. Tomporowski . 2012. “The Influence of Exercise‐Induced Fatigue on Cognitive Function.” Journal of Sports Sciences 30, no. 9: 841–850. 10.1080/02640414.2012.675083. 22494399

[fsn370304-bib-0058] Peterson, L. W. , and D. Artis . 2014. “Intestinal Epithelial Cells: Regulators of Barrier Function and Immune Homeostasis.” Nature Reviews. Immunology 14, no. 3: 141–153. 10.1038/nri3608. 24566914

[fsn370304-bib-0059] Pingitore, A. , G. P. Lima , F. Mastorci , A. Quinones , G. Iervasi , and C. Vassalle . 2015. “Exercise and Oxidative Stress: Potential Effects of Antioxidant Dietary Strategies in Sports.” Nutrition 31, no. 7–8: 916–922. 10.1016/j.nut.2015.02.005. 26059364

[fsn370304-bib-0060] Rao, Y. L. , B. Ganaraja , T. Joy , M. M. Pai , S. D. Ullal , and B. V. Murlimanju . 2020. “Neuroprotective Effects of Resveratrol in Alzheimer's Disease.” Frontiers in Bioscience (Elite Edition) 12, no. 1: 139–149. 10.2741/e863. 31585875

[fsn370304-bib-0061] Ratz‐Łyko, A. , and J. Arct . 2019. “Resveratrol as an Active Ingredient for Cosmetic and Dermatological Applications: A Review.” Journal of Cosmetic and Laser Therapy 21, no. 2: 84–90. 10.1080/14764172.2018.1469767. 29737899

[fsn370304-bib-0062] Rauf, A. , M. Imran , M. S. Butt , M. Nadeem , D. G. Peters , and M. S. Mubarak . 2018. “Resveratrol as an Anti‐Cancer Agent: A Review.” Critical Reviews in Food Science and Nutrition 58, no. 9: 1428–1447. 10.1080/10408398.2016.1263597. 28001084

[fsn370304-bib-0063] Ruhee, R. T. , and K. Suzuki . 2024. “The Immunomodulatory Effects of Sulforaphane in Exercise‐Induced Inflammation and Oxidative Stress: A Prospective Nutraceutical.” International Journal of Molecular Sciences 25, no. 3: 1–13. 10.3390/ijms25031790. PMC10855658 38339067

[fsn370304-bib-0064] Ryan, M. P. , and J. T. Pembroke . 2018. “Brevundimonas Spp: Emerging Global Opportunistic Pathogens.” Virulence 9, no. 1: 480–493. 10.1080/21505594.2017.1419116. 29484917 PMC5955483

[fsn370304-bib-0065] Shahcheraghi, S. H. , F. Salemi , S. Small , et al. 2023. “Resveratrol Regulates Inflammation and Improves Oxidative Stress via Nrf2 Signaling Pathway: Therapeutic and Biotechnological Prospects.” Phytotherapy Research 37, no. 4: 1590–1605. 10.1002/ptr.7754. 36752350

[fsn370304-bib-0066] Sitkin, S. , and J. Pokrotnieks . 2019. “Clinical Potential of Anti‐Inflammatory Effects of Faecalibacterium Prausnitzii and Butyrate in Inflammatory Bowel Disease.” Inflammatory Bowel Diseases 25, no. 4: e40–e41. 10.1093/ibd/izy258. 30085080

[fsn370304-bib-0067] Slack, J. , H. I. Noh , L. Ledbetter , and T. A. Albrecht . 2024. “The Association Between the Gut Microbiome and Fatigue in Individuals Living With Cancer: A Systematic Review.” Support Care Cancer 32, no. 4: 267. 10.1007/s00520-024-08468-5. 38575690

[fsn370304-bib-0068] Tan, W. , K. Q. Yu , Y. Y. Liu , et al. 2012. “Anti‐Fatigue Activity of Polysaccharides Extract From Radix Rehmanniae Preparata.” International Journal of Biological Macromolecules 50, no. 1: 59–62. 10.1016/j.ijbiomac.2011.09.019. 21983027

[fsn370304-bib-0069] Tian, B. , and J. Liu . 2020. “Resveratrol: A Review of Plant Sources, Synthesis, Stability, Modification and Food Application.” Journal of the Science of Food and Agriculture 100, no. 4: 1392–1404. 10.1002/jsfa.10152. 31756276

[fsn370304-bib-0070] Wang, C. , H. Zhu , Y. Cheng , Y. Guo , Y. Zhao , and H. Qian . 2023. “Aqueous Extract of *Brassica rapa* L.'s Impact on Modulating Exercise‐Induced Fatigue via Gut‐Muscle Axis.” Nutrients 15, no. 22: 1–20. 10.3390/nu15224737. PMC10674577 38004133

[fsn370304-bib-0071] Williams, J. H. , T. W. Batts , and S. J. Lees . 2013. “Reduced Muscle Glycogen Differentially Affects Exercise Performance and Muscle Fatigue.” International Scholarly Research Notices 2013: 1–8.

[fsn370304-bib-0072] Wu, R. E. , W. C. Huang , C. C. Liao , Y. K. Chang , N. W. Kan , and C. C. Huang . 2013. “Resveratrol Protects Against Physical Fatigue and Improves Exercise Performance in Mice.” Molecules 18, no. 4: 4689–4702. 10.3390/molecules18044689. 23603951 PMC6270062

[fsn370304-bib-0073] Xu, M. , R. Liang , Y. Li , and J. Wang . 2017. “Anti‐Fatigue Effects of Dietary Nucleotides in Mice.” Food & Nutrition Research 61, no. 1: 1334485. 10.1080/16546628.2017.1334485. 28659748 PMC5475326

[fsn370304-bib-0074] Xu, Z. , X. Sun , B. Ding , M. Zi , and Y. Ma . 2023. “Resveratrol Attenuated High Intensity Exercise Training‐Induced Inflammation and Ferroptosis via Nrf2/FTH1/GPX4 Pathway in Intestine of Mice.” Turkish Journal of Medical Sciences 53, no. 2: 446–454. 10.55730/1300-0144.5604. 37476875 PMC10387861

[fsn370304-bib-0075] Young, A. , C. Oldford , and R. J. Mailloux . 2020. “Lactate Dehydrogenase Supports Lactate Oxidation in Mitochondria Isolated From Different Mouse Tissues.” Redox Biology 28: 101339. 10.1016/j.redox.2019.101339. 31610469 PMC6812140

[fsn370304-bib-0076] Zeng, M. Y. , N. Inohara , and G. Nuñez . 2017. “Mechanisms of Inflammation‐Driven Bacterial Dysbiosis in the Gut.” Mucosal Immunology 10, no. 1: 18–26. 10.1038/mi.2016.75. 27554295 PMC5788567

[fsn370304-bib-0077] Zhang, C. J. , J. Y. Guo , H. Cheng , et al. 2020. “Spatial Structure and Anti‐Fatigue of Polysaccharide From Inonotus Obliquus.” International Journal of Biological Macromolecules 151: 855–860. 10.1016/j.ijbiomac.2020.02.147. 32068062

[fsn370304-bib-0078] Zhang, L. X. , C. X. Li , M. U. Kakar , et al. 2021. “Resveratrol (RV): A Pharmacological Review and Call for Further Research.” Biomedicine & Pharmacotherapy 143: 112164. 10.1016/j.biopha.2021.112164. 34649335

[fsn370304-bib-0079] Zhang, N. , X. Mao , R. W. Li , et al. 2017. “Neoagarotetraose Protects Mice Against Intense Exercise‐Induced Fatigue Damage by Modulating Gut Microbial Composition and Function.” Molecular Nutrition & Food Research 61, no. 8: 1–34. 10.1002/mnfr.201600585. 28083922

[fsn370304-bib-0080] Zhang, X. , S. Jing , H. Lin , et al. 2019. “Anti‐Fatigue Effect of Anwulignan via the NRF2 and PGC‐1α Signaling Pathway in Mice.” Food & Function 10, no. 12: 7755–7766. 10.1039/c9fo01182j. 31696200

[fsn370304-bib-0081] Zhang, Y. , G. Yang , Y. Gao , et al. 2024. “Total Minor Ginsenosides Exert Anti‐Fatigue Effects via Antioxidant, Anti‐Inflammatory, Regulating Gut Microbiota and Serum Metabolism.” Life Sciences 359: 123231. 10.1016/j.lfs.2024.123231. 39537101

[fsn370304-bib-0082] Zhao, R. , R. Wu , J. Jin , et al. 2023. “Signaling Pathways Regulated by Natural Active Ingredients in the Fight Against Exercise Fatigue—A Review.” Frontiers in Pharmacology 14: 1269878. 10.3389/fphar.2023.1269878. 38155906 PMC10752993

[fsn370304-bib-0083] Zhong, L. , L. Zhao , F. Yang , W. Yang , Y. Sun , and Q. Hu . 2017. “Evaluation of Anti‐Fatigue Property of the Extruded Product of Cereal Grains Mixed With Cordyceps Militaris on Mice.” Journal of the International Society of Sports Nutrition 14: 15. 10.1186/s12970-017-0171-1. 28588427 PMC5457539

[fsn370304-bib-0084] Zhou, D. D. , M. Luo , S. Y. Huang , et al. 2021. “Effects and Mechanisms of Resveratrol on Aging and Age‐Related Diseases.” Oxidative Medicine and Cellular Longevity 2021: 9932218. 10.1155/2021/9932218. 34336123 PMC8289612

[fsn370304-bib-0085] Zhou, Q. , Y. Wang , X. Han , S. Fu , C. Zhu , and Q. Chen . 2022. “Efficacy of Resveratrol Supplementation on Glucose and Lipid Metabolism: A Meta‐Analysis and Systematic Review.” Frontiers in Physiology 13: 795980. 10.3389/fphys.2022.795980. 35431994 PMC9009313

[fsn370304-bib-0086] Zhou, S. S. , and J. G. Jiang . 2019. “Anti‐Fatigue Effects of Active Ingredients From Traditional Chinese Medicine: A Review.” Current Medicinal Chemistry 26, no. 10: 1833–1848. 10.2174/0929867324666170414164607. 28413958

[fsn370304-bib-0087] Zivarpour, P. , Ž. Reiner , J. Hallajzadeh , and L. Mirsafaei . 2022. “Resveratrol and Cardiac Fibrosis Prevention and Treatment.” Current Pharmaceutical Biotechnology 23, no. 2: 190–200. 10.2174/1389201022666210212125003. 33583368

